# Gestational Pemphigoid Presenting in the Second Trimester of Pregnancy: A Rare Finding

**DOI:** 10.7759/cureus.25531

**Published:** 2022-05-31

**Authors:** Rabia Ghafoor, Iqra M Hanif, Muhammad A Ullah, Aisha M Husseni

**Affiliations:** 1 Dermatology, Jinnah Sindh Medical University, Jinnah Postgraduate Medical Centre, Karachi, PAK; 2 Dermatology, Jinnah Postgraduate Medical Centre, Karachi, PAK; 3 Medicine, Jinnah Sindh Medical University, Jinnah Postgraduate Medical Centre, Karachi, PAK; 4 Internal Medicine, Jinnah Postgraduate Medical Centre, Karachi, PAK

**Keywords:** steroids, pregnancy, blisters, autoimmune, gestational pemphigoid

## Abstract

Gestational pemphigoid (GP) is a rare autoimmune blistering disorder, occurring in 1 in 60,000 pregnancies. It occurs in the second or third trimester of pregnancy and is characterized by autoantibodies against hemidesmosomal proteins. A variety of dermatological conditions are associated with pregnancy; among these skin diseases, gestational pemphigoid is very rare. The purpose of this report is to highlight the specific findings of this rare disease to enable clinicians to take prompt intervention in treating this condition. A 23-year-old multigravida, with no known comorbidities, presented to us at 18 weeks of gestation with complaints of intensely pruritic tense blisters all over the body, sparing the scalp, palms, and soles. The diagnosis was confirmed by skin biopsy for histopathology, after which the patient was started on prednisolone, which was then gradually tapered to an appropriate maintenance dose and then discontinued as the patient did not report any new lesions after delivery. Gestational pemphigoid can recur in subsequent pregnancies with more severe lesions. However, this was the first time the patient presented with this condition in her third pregnancy. Proper management of this disease requires close monitoring and appropriate drug therapy to reduce maternal and neonatal morbidity.

## Introduction

Pregnancy-related skin diseases are classified into gestational pemphigoid (GP), polymorphic eruption of pregnancy, intrahepatic cholestasis of pregnancy, and atopic eruption of pregnancy [1]. GP is a rare, autoimmune blistering disorder that presents analogous to the pemphigoid group of blistering disorders. Its prevalence in the general population is estimated to be 1 in 60,000 pregnancies [2]. Its pathophysiology is similar to that of the general pemphigoid disorders, which is characterized by autoantibodies targeting hemi-desmosomal proteins BP 180 and BP 230 at the dermo-epidermal junction [2]. It is also thought to be associated with changes in progestin during pregnancy [3].

This condition usually presents in the second or third trimester of pregnancy with intense abdominal itching and urticarial plaques followed by blistering [3]. The purpose of this report is to highlight the specific findings of this rare disease to enable clinicians to make prompt interventions in treating this condition.

## Case presentation

**Figure 1:**
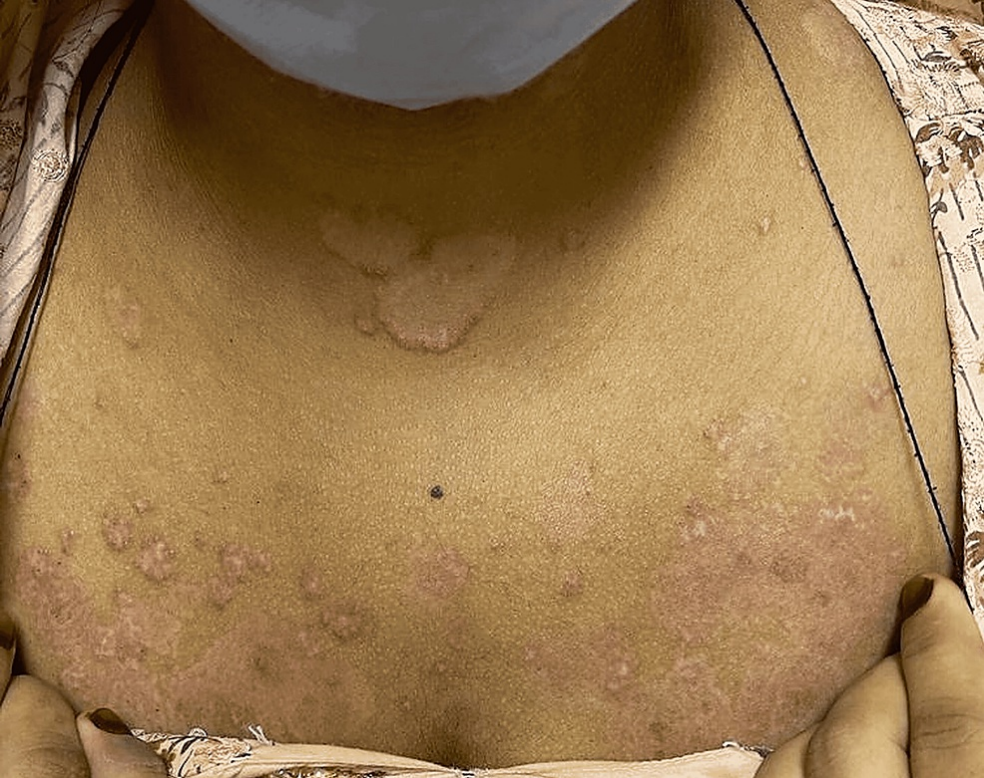
Multiple scattered erosions on the chest.

**Figure 2:**
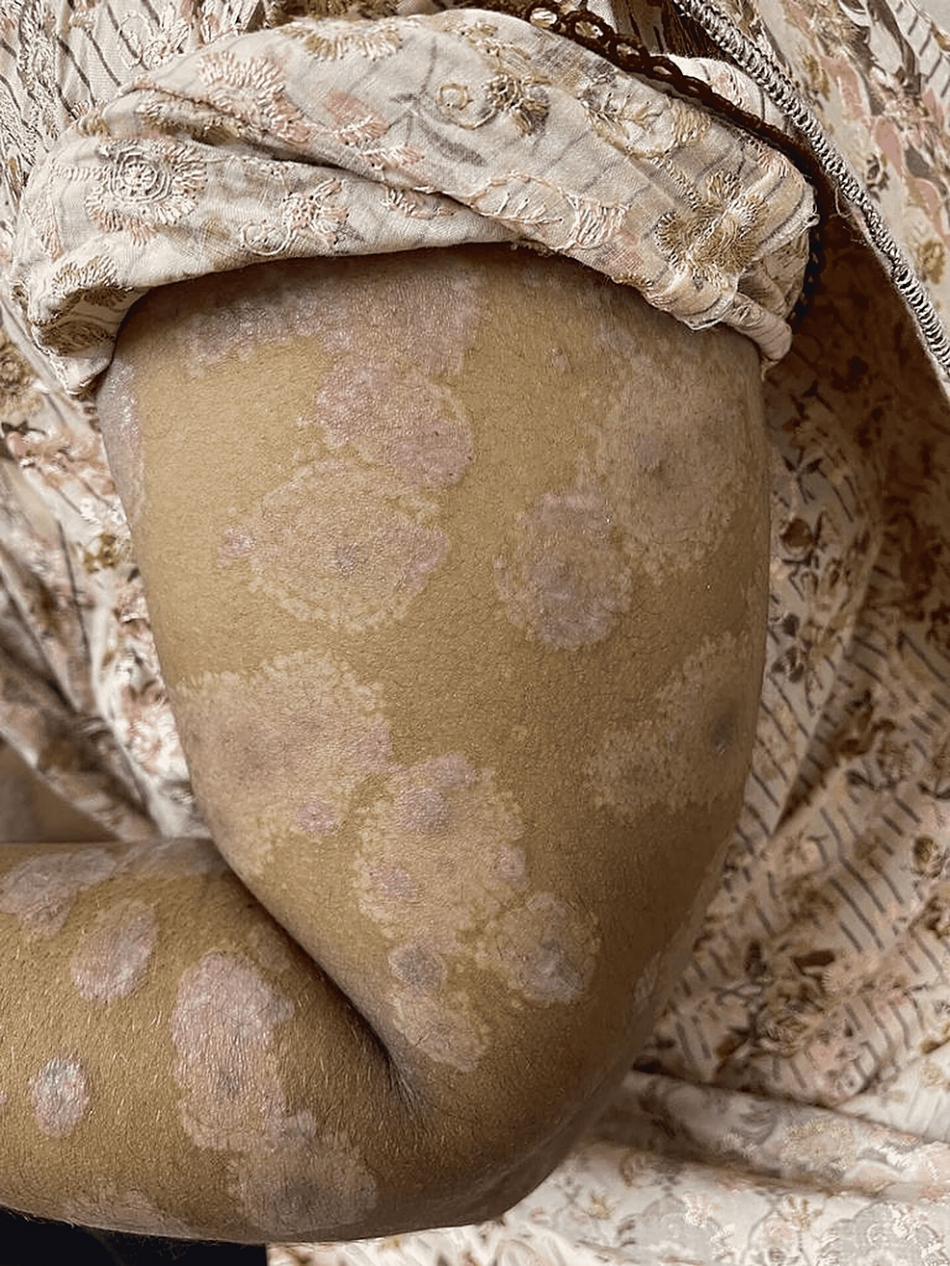
Multiple erosions on left arm.

**Figure 3:**
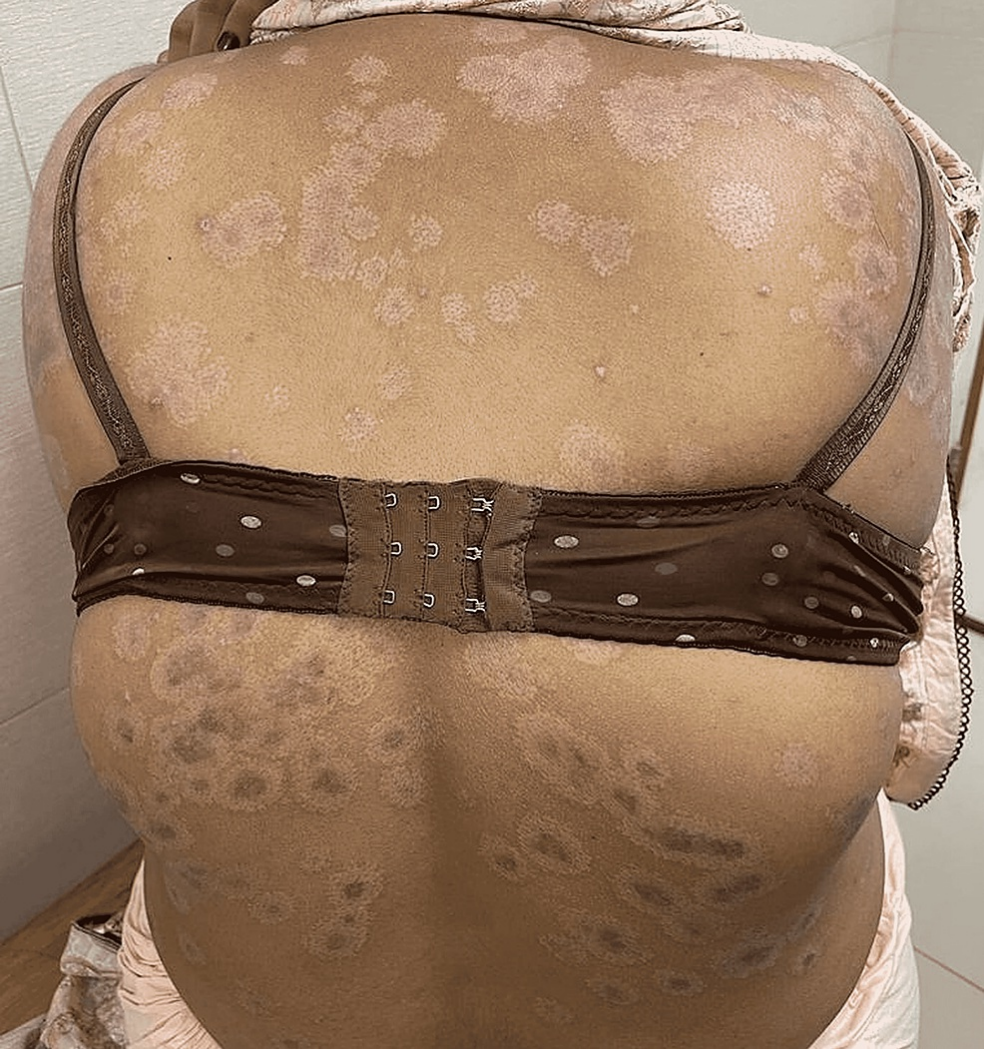
Widespread erosions on back.

**Figure 4:**
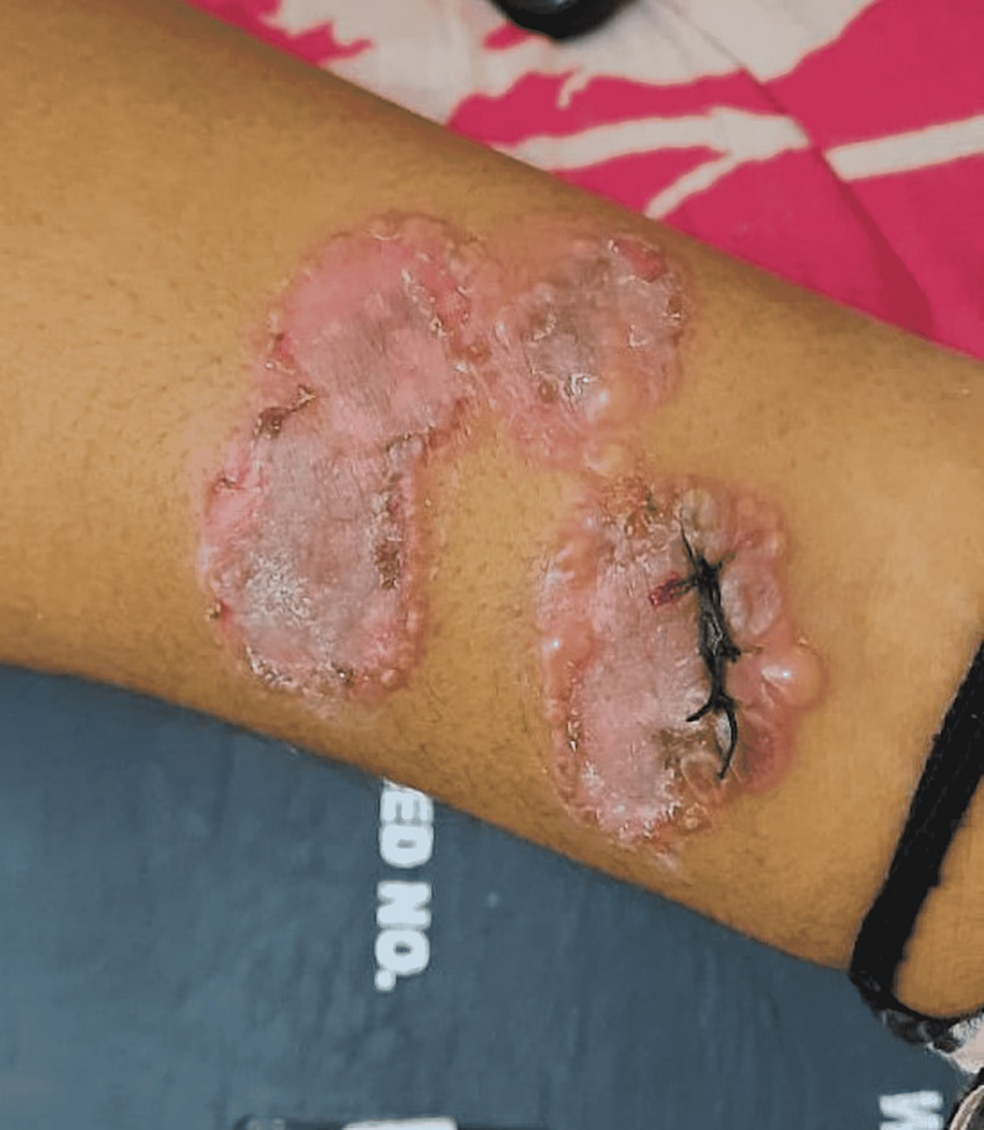
Erosions and new blister on right arm. Biopsy sample was taken from this blister which was then sutured.

A 23-year-old multigravida, with no known comorbidities, was admitted to the Dermatology department of Jinnah Postgraduate Medical Centre, Karachi, Pakistan. The patient, who has two children and suffered a miscarriage three years back, presented to us at 18 weeks of gestation with complaints of tense fluid-filled blisters and itching for the past 20 days. The lesions first appeared on both arms, which then progressed to involve her entire body over a period of one week. There was no history of any blistering disease in previous pregnancies. On examination, the patient had multiple scattered tense blisters all over the body, including oral mucosa, which started from the abdomen but spared the scalp, palms, and soles. A few of the blisters ruptured spontaneously, forming erosions (Figures 1-3). New blisters were also seen on the right arm (Figure 4) and lips after a few days of admission.

**Figure 5:**
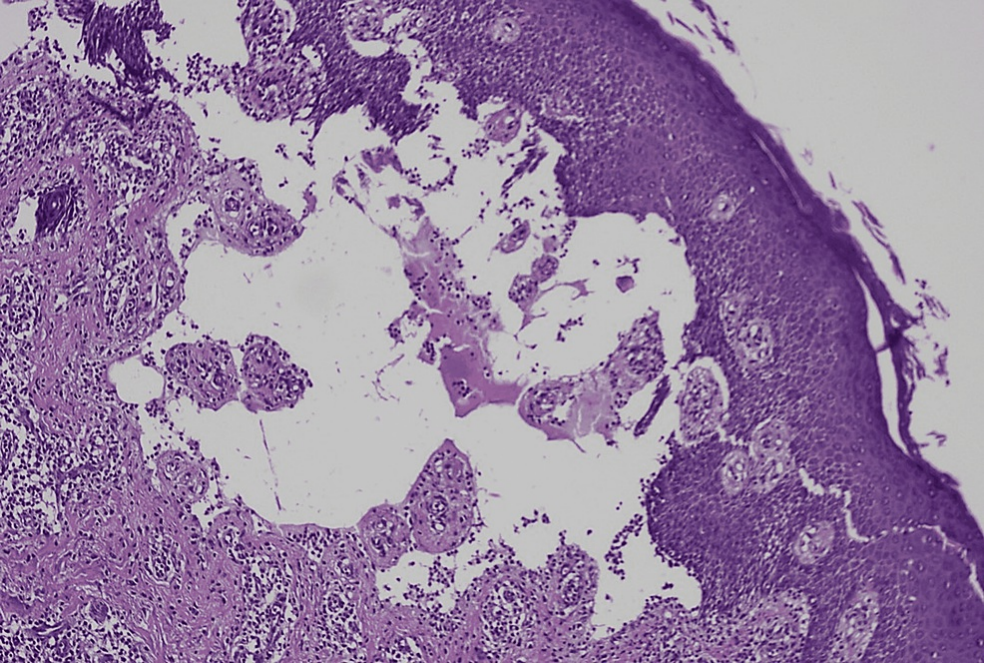
Histopathological picture using hematoxylin and eosin stain showing sub-epidermal vesicles. Magnification ×10.

**Figure 6:**
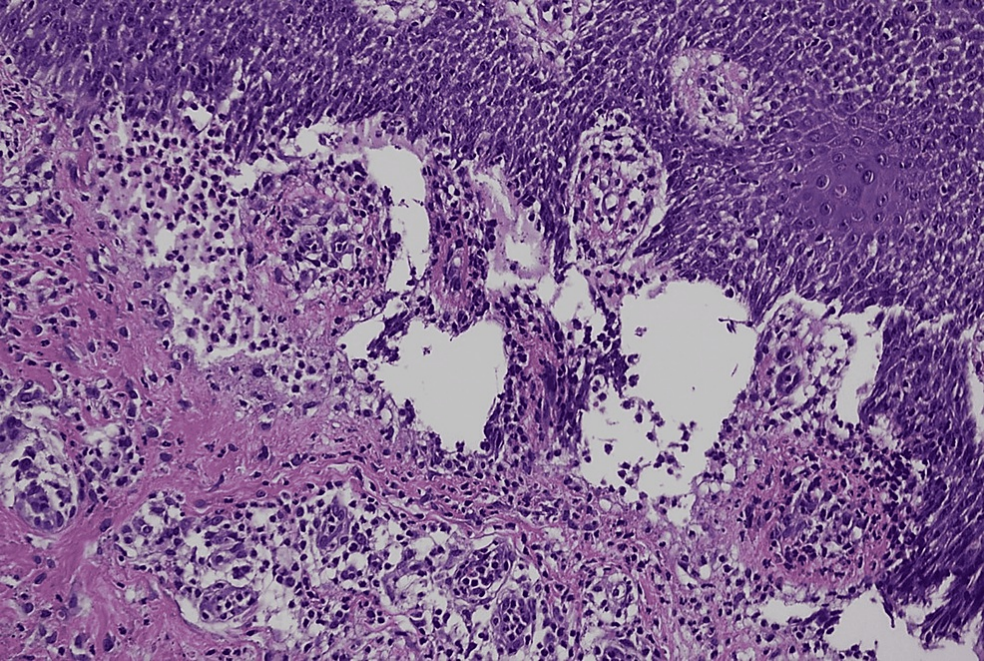
Histopathological picture using hematoxylin and eosin stain showing spongiosis. Magnification ×20.

**Figure 7:**
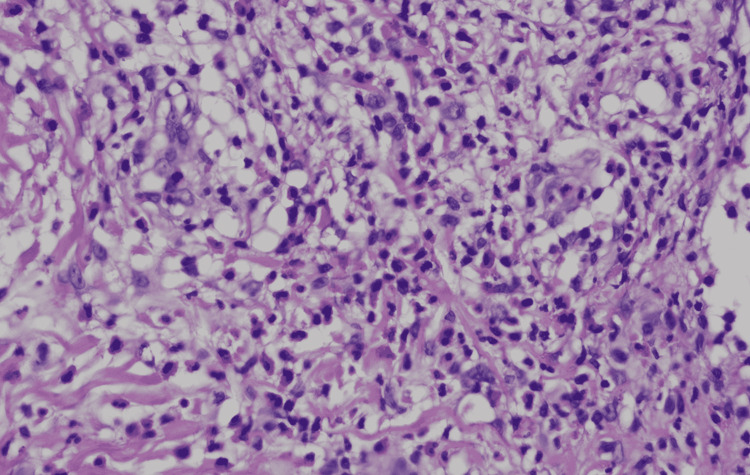
Histopathological picture using hematoxylin and eosin stain showing lympho-eosinophilic infiltrates. Magnification ×40.

**Figure 8:**
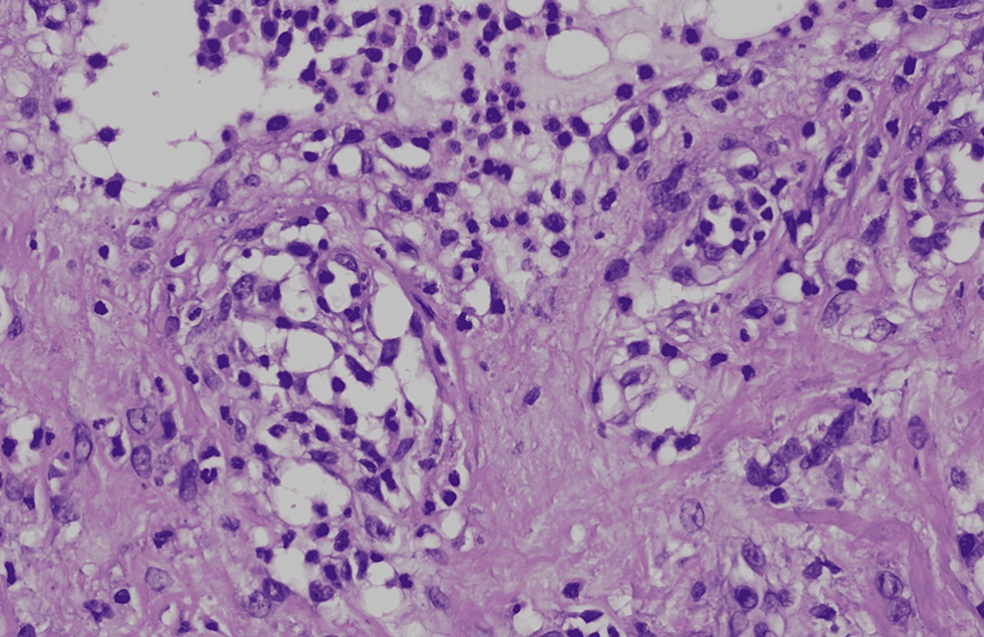
Histopathological picture using hematoxylin and eosin stain showing lympho-eosinophilic infiltrates. Magnification ×40.

When she presented herself to us, she had been receiving undocumented treatments from different doctors. Initial baseline investigations, including complete blood count, urea, creatinine, electrolytes, C-reactive protein, erythrocyte sedimentation rate, and liver function tests, were normal. On general physical examination, the abdomen was soft and distended proportional to gestational age, along with normal cardiovascular and respiratory examination. Her vitals on multiple assessments were also normal. Differential diagnoses of gestational pemphigoid and bullous drug eruptions were established. The diagnosis was confirmed by a skin biopsy for histopathology, which showed sub-epidermal vesicles (Figure 5) with spongiosis (Figure 6) and lympho-eosinophilic infiltrates (Figures 7-8).

However, direct immunofluorescence (DIF) was negative. The patient was admitted and started on topical and oral prednisolone 30 mg/day and loratadine. She responded well to the treatment and no new blisters were observed, thus the steroids were gradually tapered. She was then discharged after five days and followed in at the outpatient clinic. The patient delivered a completely healthy baby.

## Discussion

Gestational pemphigoid shares similar clinical features with most autoimmune blistering disorders. However, its presentation in pregnancy makes it a rare cutaneous disorder. Amongst the various cutaneous disorders observed in pregnancy in Pakistan, gestational pemphigoid was the least observed condition [4], and in another study, no case of GP was observed [5]. Patients with gestational pemphigoid are also at increased risk of developing other autoimmune disorders [3].

The lesions usually involve the abdomen, trunk, and limbs, sparing the face and mucous membranes [1], but this patient also had both facial and mucosal involvement. Thus, gestational pemphigoid has an irregular presentation, and its early detection is very crucial in its prognosis.

There are various ways to confirm the diagnosis of autoimmune blistering disorders [6,7]. We did a detailed workup, including clinical and histological correlations. Two punch biopsies were taken for histopathological and DIF analysis. The DIF was negative but the hematoxylin and eosin staining revealed sub-epidermal vesicles with lymphoeosinophilic infiltration and spongiosis, which formed the basis of our diagnosis. Baseline investigations, including a complete blood count, serum electrolytes, and hepatic and renal profile, were normal.

After the biopsy was done, she was started on prednisolone 30 mg/day at 18 weeks. The dose was maintained at the same rate as no new lesions developed. This treatment protocol has been followed multiple times, and like this patient, it proved to be helpful in alleviating the symptoms [8]. Topical corticosteroids and antihistamines were also added to provide symptomatic relief. Significant improvement of the lesions (post-inflammatory macules) was observed during the two-week follow-up and her steroids were gradually tapered.

Gestational pemphigoid can recur in subsequent pregnancies with more severe lesions during menstruation and ovulation [9]. However, in this case, this was the first time this patient presented with this condition in her third pregnancy that was easily controlled by treatment and did not reoccur.

In terms of pregnancy outcomes, there have been reports of preterm delivery and fetal growth restriction as associated risk factors for GP [9,10]. About 10% of infants have also developed mild skin lesions due to placental transfer of antibodies that heal after a few days, but overall the outcomes are good. In this case, the patient delivered a healthy female weighing around 8 pounds via cesarean section. The patient did not report any new lesions after the delivery.

## Conclusions

Gestational pemphigoid is a rare disease but associated with adverse fetal outcomes. Therefore, it is very important to identify and treat it in its initial stages to avoid complications in the pregnancy. A comprehensive collaboration between the dermatology and the obstetrics and gynecology departments is important for accurate diagnosis and treatment.
